# GermiX: A skin friendly hand sanitizer with prolonged effectivity against pathogenic bacteria

**DOI:** 10.1186/s13568-020-01151-y

**Published:** 2020-12-01

**Authors:** Acharya Balkrishna, Kanchan Singh, Hoshiyar Singh, Swati Haldar, Anurag Varshney

**Affiliations:** 1 Drug Discovery and Development Division, Patanjali Research Institute (Governed By Patanjali Research Foundation Trust), Roorkee-Haridwar Road, Haridwar, Uttarakhand 249405 India; 2 Department of Allied and Applied Sciences, University of Patanjali, NH-58, Haridwar, Uttarakhand 249405 India

**Keywords:** Patanjali hand sanitizer, Prolonged surface retentivity, Hand sanitizer, Cost-effective

## Abstract

COVID-19 pandemic has almost made hand sanitization a ritual resulting in a steep increase in the frequency of hand sanitization and an unprecedented surge in demand for hand sanitizers. In fact, several governments had to ration hand sanitizers in the retail outlets and over the counter chemist shops. Additionally, Indian government has put a cap on the prices of hand sanitizers. Currently, large sections of global and Indian population are grappling under financial crises. Therefore, mandatory hand sanitization has made an unwelcoming, yet unavoidable addition to the already-hard-to-maintain-grocery-list. Here, we have compared the anti-microbial efficacy of Patanjali Hand Sanitizer (PHS), developed and marketed by Patanjali Ayurved Ltd. (an India-based food and herbal medicine company) with one of the topmost hand sanitizers currently used under clinical set-ups. PHS has anti-microbial efficacy comparable to that of the standard hand sanitizer. Besides, disc diffusion and time-dependent thumb print assays showed that PHS has longer retentivity on the applied surfaces, suggesting lesser consumption of the sanitizer and concomitant relaxation on the monthly grocery budget. Observed anti-bacterial potency of PHS is attributed to the disruption of bacterial cell membrane, as employed by alcohol-based hand sanitizers. A rough estimation revealed that PHS is ~ 4.3 times cost effective than the standard hand sanitizer used as the positive control in this study. Taken together, PHS is a suitable alternative for existing hand sanitizers available in the market that can relax the demand–supply strain and soften significantly the burden of monthly expenditure on hand sanitizers.

## Introduction

Hand sanitizers are one of the effective anti-microbial and anti-viral in the form of foam, liquids and gels. Now days the hand sanitizer play a very important role in maintaining the hand hygiene by denaturation and coagulation of microbial proteins resulting in cell lysis. Alcohols, are the one of the most effective agents for reducing the number of viable pathogens on the hands. Hands are the major mode of transmission of infectious microbes from one person to another (WHO Guidelines on Hand Hygiene in Health Care 2009). According to the Center for Disease control and prevention (CDC) approximately 2 million people got affected by hospital acquired infection per year. The public health sector like CDC and WHO suggest to use hand sanitizer for preventing spreading of infectious microbes through hands (WHO Guidelines on Hand Hygiene in Health Care 2009).

With the main focus being on prevention of spreading of SARS-COV-2 coronavirus infection, hand sanitization has become the mainstay of the COVID-19 mediated altered lifestyle. Hand sanitizers are alcohol based. So, they do not remain on the hands for long after application, thus, necessitating very frequent sanitization. However, a formulation that can effectively prolong the retention time of the sanitizer after its application on the hands will reduce the requirement of very frequent hand sanitization. This, in turn, would lead to lesser consumption of the hand sanitizer and eventually, less burden on the pocket. Besides, with the swelling demand for hand sanitizer, it is also becoming challenging to keep up the supply (https://losangeles.cbslocal.com/2020/04/07/ceo-warns-supply-chain-issues-could-halt-hand-sanitizer-production-two-weeks/). Therefore, a hand sanitizer with anti-microbial efficacy equivalent to that of the existing ones in the market but with added advantage of longer post-application retention time will help in (a) cope up with the ever increasing demand for hand sanitizer by facilitating lesser consumption, and (b) reducing the economic burden on the masses by decreasing the per head consumption.

Here, we have verified the cytosafety and anti-microbial efficacy of a semi-herbal hand sanitizer in comparison to a standard hand sanitizer used in clinical set-ups. We chose this positive control to bring forth the functional robustness of the semi-herbal hand sanitizer. We have also discussed a projected reduction in the financial load that the use of the semi-herbal hand sanitizer can facilitate. This is particularly to provide a comparative perspective.

## Materials and methods

### Bacterial strains, cell lines and chemicals

Skin pathogenic *Staphylococcus epidermidis* (MTCC435) and *Staphylococcus aureus* (MCC2408) were procured from Microbial Type Culture Collection (MTCC) (CSIR-Institute of Microbial Technology, Chandigarh, India). Human squamous epithelial skin cell line (A-431) was procured from American Type Culture Collection (ATCC) licensed repository at National Centre for Cell Sciences (NCCS, Pune, India). Bacterial growth media were procured from Difco (Fisher Scientific, USA). Cell culture growth media and supplements were purchased from Gibco (Thermo Fisher Scientific, USA). All other chemicals used in this study were obtained from Sigma Aldrich (Missouri, USA), unless otherwise stated. The test sample is a semi-herbal hand sanitizer, developed and manufactured by Patanjali Ayurved Ltd., Haridwar, India, with the trade name “Germix”. Henceforth, it will be referred as Patanjali Hand Sanitizer (PHS). PHS is composed of a blend of medicinal herbs like *Azadirachta indica* [common name: Neem (Hindi), Indian lilac (English)], *Ocimum sanctum* [common name: Tulsi (Hindi), Holy Basil (English)] and *Aloe barbadensis* [common name: Ghritkumari (Hindi), Aloe vera (English)] and iso-propyl alcohol along with other excipients, fragrances (Neem and Tulsi), coloring agents and preservatives. PHS was sourced from Patanjali Megastore, Haridwar (Batch. No. AAGE001, Expiry date: 02/2022) manufactured as per the license no. Uttra.Ayu-181/2009 issued by Licensing Authority, Ayurvedic and Unani Services, Uttarakhand, Dehradun, India.

### Activation of microbial culture

*S. epidermidis* and *S. aureus* were reactivated from glycerol stocks prior to susceptibility testing by streaking a loopful of frozen stock on nutrient agar plate. Subsequently, liquid cultures were set up in test tubes by inoculating single colonies from the above plates in nutrient broths and incubated overnight at 37 °C. All experiments were conducted with these liquid cultures.

### Antibacterial activity by disc diffusion assay

Culture plates were prepared by pouring 20 ml sterilized Muller Hinton Agar (MHA) into pre-sterilized bacteriological petri dishes. 0.1 ml [inoculum sizes were adjusted by using 0.5 Mcfarland standard with approximately 1.0 × 10^8^ colony forming units (CFU/mL)] of inoculum suspension of each bacterial strain was spread uniformly over the agar medium using sterile glass spreaders. 6 mm sterile standard filter discs were either soaked with 40 µl PHS or standard hand sanitizer (positive control) and placed on the bacterial spreads in the above agar plates. The plates were incubated at 37 °C for 24 h. The diameters of zones of inhibition (mm) were measured and recorded. Experiments were carried out in triplicates.

### Determination of minimum inhibitory concentration (MIC)

96-well micro plates were used to determine the MIC of PHS against pathogenic *S. epidermidis* and *S. aureus*. PHS was two-fold serially diluted to obtain final percent volume/volume (% v/v) concentration range of 0.01 to 25% after adding 75 µl of suspension of desired bacterial strain prepared in MHB (containing 10^8^ CFU/mL) to a final volume of 150 μl per well. The plates were then incubated for 24 h at 37 °C with shaking at 180 rpm. Following incubation, MIC values were quantified by measuring absorbance at 600 nm in a microplate reader (Envision, Perkin Elmer). An identical concentration range of standard hand sanitizer was used as positive control for comparison. Data is represented graphically as mean ± SE of percent (%) inhibition calculated from three independent experiments using Graphpad prism (version 7.0). MIC_50_ values of PHS and positive control for *S. epidermidis* and *S. aureus* were calculated using inbuilt options provided with Graphpad prism. Dose response curves for PHS and positive control for *S. epidermidis* and *S. aureus* were also plotted indicating the respective MIC_50_ and Hill slope values.

### Determination of anti-microbial efficacy through thumb print assay

A total of 30 volunteers participated in this study. Thumb impressions of each volunteer were taken on sterile tryptic soya agar plates before and after washing with water or sanitizing with standard sanitizer (positive control) or PHS. The plates were then incubated under aerobic conditions in a bacteriological incubator at 37 °C for 24 h. Antimicrobial potencies of the sanitizers were calculated from the number of visible microbial colony present before and after application of hand sanitizers or water using the following formula

$$\% {\text{ potency }} = \, \left( {{\text{BBW}} - {\text{BAW}}} \right)/{\text{BBW}}*{1}00$$

where BBW and BAW, stand for bacterial load before and after water wash or hand sanitization, respectively.

In order to check the prolonged anti-microbial effect of PHS, we conducted as similar experiment with kind re-participation of three of the above volunteers. In this experimental set up we collected the thumb prints from left and right hands of these volunteers on tryptic soya a gar plates before sanitization. Since, all the volunteers were right-handed, we chose to sanitize the left thumb with the commercial sanitizer and the right thumbs with PHS and immediately, collected the thumb prints on agar plates. The volunteers were allowed to get back to their works and freely use their hands before, taking their thumb prints after 30 min, this time without sanitization. Likewise, another set of prints were collected after 60 min all this while, allowing the volunteers to freely use their hands. The rationale behind choosing right thumb for PHS was to subject it to a more robust testing conditions, given the volunteers were right handed.

### Evaluation of bacterial membrane damage through potassium efflux assay

The amount of potassium (K^+^) ions leaking is a quantitative manifestation of the extent of membrane damage experienced by the bacterial cells due any kind of bactericidal treatment. Potassium efflux was estimated according to a previously described method (Rajawat et al. 2014). Briefly, 5 ml overnight cultures of *S. epidermidis* and *S. aureus* were washed twice with PBS (pH 7.0) and diluted to obtain 10^8^ CFU/ml. 2× MIC_50_ of PHS or standard hand sanitizer was added to the bacterial solution. Samples were collected immediately after adding the test articles (0 min) and subsequently after incubating for 1, 3, and 5 min at 37 °C with stirring. Collected samples were centrifuged at 8000 rpm for 10 min, and supernatant incubated with sodium cobalt nitrite [Na_3_Co(NO_2_)_6_] at 37 °C for 45 min to accomplish the maximum precipitation of potassium, following which the mixture was centrifuged at 13,000 rpm for 5 min to settle the precipitate. Obtained precipitate was collected and washed twice with distilled water and once with absolute ethanol. After careful washing, 10 mL of concentrated HCl was added and incubated at 37 °C for 15–20 min to develop color. Finally, absorbance was measured at 623 nm. Similarly known dilutions of KCl were processed for standard curve preparation.

### Cytotoxicity assay

A-431 cells were cultured and maintained in high glucose DMEM medium supplemented with 10% Fetal bovine serum along with 100 U/ml Penicillin, 100 μg/ml Streptomycin, in a humidified incubator at 37 °C with 5% CO_2_. Cytotoxicity of hand sanitizers was evaluated according to a previously published protocol (Hall et al. 2009). Briefly, cells were plated in 96 well plate at density of 10,000 cells/well in complete medium. Cells were incubated for 3 days until a confluent monolayer was formed. Hand sanitizers were diluted with complete medium to prepare different % v/v concentrations (25, 12.5, 6.25, 3.125, and 1.562) and added to cells for 2 and 5 min. Subsequently, the hand sanitizers were washed out using pre-warmed 1× phosphate buffer saline (PBS). Fresh medium was added to cells and incubated for 24 h in a humidified CO_2_ incubator. Viable cells were stained for 3 h at 37 °C with alamar blue (15 µg/ml) prepared in serum free DMEM. Fluorescence from each well was measured using PerkinElmer Envision microplate reader with excitation and emission at 560 and 590 nm, respectively. Percentage cell viability was calculated with respect to untreated cells and data represented as mean ± SE.

## Results

### Sanitizing efficacy of the semi-herbal hand sanitizer is comparable to the commercially available hand sanitizer

**Fig. 1:**
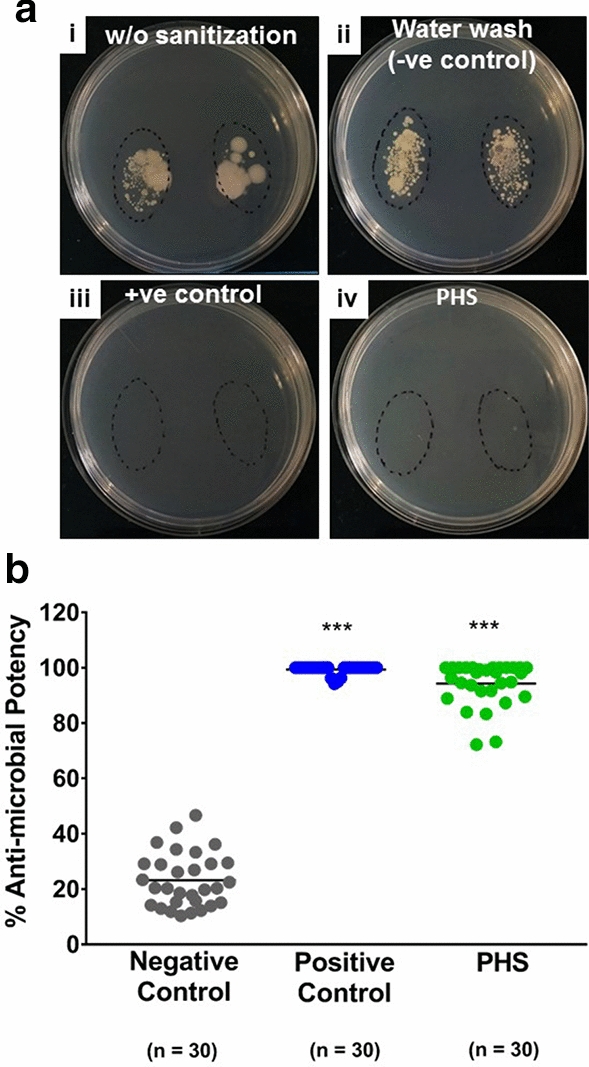
Sanitizing Efficacy of Patanjali Hand Sanitizer (PHS). **a** Representative digital images of bacteriological plates with thumb print from the same volunteer before (i) and after sanitization with either standard hand sanitizer (+ ve control) (ii) or PHS (iv). Plate image with thumb prints after washing with water is taken as –ve control (ii). **b** Sanitization efficacy is represented as % potency through a scatter plot. Statistical significance was determined through one-way ANNOVA employing Tukey’s multiple comparison testing and observation represented as ***p < 0.001, when significantly different relative to the negative control

We conducted a comparative evaluation of the sanitizing potency of PHS. We chose a well-established alcohol based hand sanitizer that has been in commercial use for past 50 years in various clinical and laboratory set ups as positive control to be used as a standard to compare the efficacy of PHS. To avoid any conflict of interest, we are deliberately not disclosing the name of the positive control here. Thumb prints of volunteers (n = 30) were collected before and after sanitization with either positive control or herbal hand sanitizer. Thumb prints collected from the same volunteers after washing with water were included as experimental (negative) control to ensure the authenticity of the methodology employed. Sanitization potency was determined based on the number of colonies that grew before and after sanitization in each case. Representative digital images of the plates with unwashed or without sanitization thumb prints showed the starting microbial load for each volunteer (Fig. 1a(i)). Washing with water did not reduce the microbial load much (Fig. 1a(ii)). Rubbing thumbs with the standard sanitizer, as expected, showed complete removal of the microbial load as evident from absence of any colony on the plate (Fig. 1a(iii)). Interestingly, rubbing thumbs with PHS was also able to remove the bacteria almost completely, exhibiting a comparable performance to that of the standard hand sanitizer (Fig. 1a(iv)). Sanitization efficacy was quantitatively perceived as percent (%) antimicrobial potency and calculated as percent reduction in colony number after rubbing the thumbs with water (for negative control), standard (for positive control) and PHS (Fig. 1b). We observed an average (± SD) 23.18 (± 9.75) % antimicrobial potency of water. Obviously, the alcohol based standard exhibited almost 100% antimicrobial potency (calculated 99.40 ± 1.60%). PHS was equally efficient in reducing the microbial load as the standard with a calculated average antimicrobial potency of 94.30 ± 7.73%. From these observations, we concluded that PHS has an antimicrobial potency comparable to one of the topmost commercial hand sanitizer available in the market.

### Prolonged anti-microbial activity of PHS

**Fig. 2:**
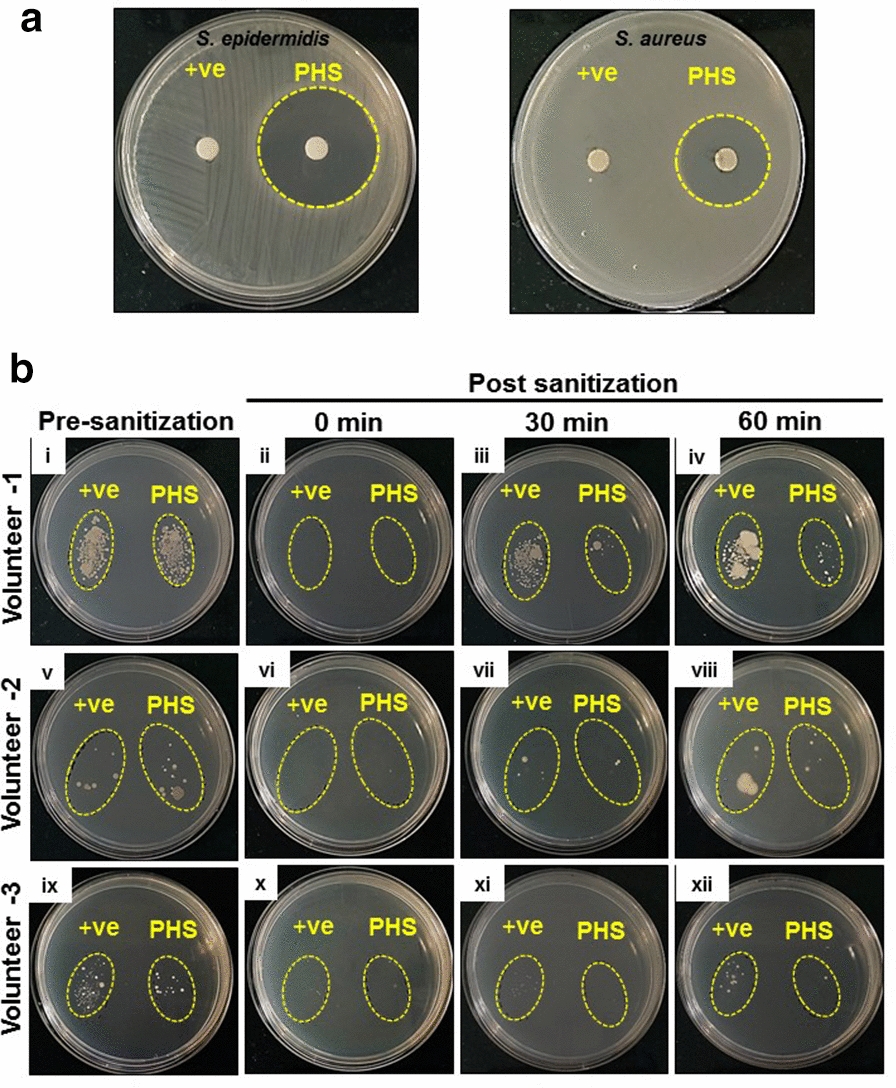
Extended surface retentivity of PHS facilitates prolonged anti-microbial activity. **a** Prolonged surface retentivity shown through representative digital images of bacteriological plates showing the zones of inhibition (demarcated with yellow broken circles) for *S. epidermidis* and *S. aureus* around the discs soaked in PHS. Discs soaked in standard hand sanitizer (+ ve control) did not have any such bacterial growth free surrounding zones. **b** Sustained anti-microbial activity resulting from protracted availability of PHS from the applied surface is represented through digital images of thumb prints containing agar plates. These prints were collected from three of the volunteers (who participated earlier) before and at 0. 30 and 60 min after sanitizing their thumbs with either standard hand sanitizer (+ ve control) or PHS

We conducted the classical disc diffusion assay to assess the anti-microbial potency of PHS. An equal volume of 40 µl of either the standard hand sanitizer or PHS was applied on 6 mm sterile filter discs and allowed to get absorbed for 5 min at room temperature. The discs were then placed on agar plates with uniformly spread *S. epidermidis* or *S. aureus* on them. The set-up was incubated for 24 h at 37 °C in a bacteriological incubator. The plates were checked for clear bacteria free zones of inhibition around the filter discs. While there was a clear zone of inhibition around the disc with PHS in case of both *S. epidermidis* and *S. aureus*, to our surprise, we did not find any such zones around the discs with standard hand sanitizer on either of the bacterial plates (Fig. 2a). Indeed, this was quite intriguing and suggested an absence of the standard sanitizer in the discs. A plausible reason for this could be quick evaporation of the sanitizer from the discs. In contrast, the gel based formulation is likely to reduce the rate of evaporation of the constituting alcohol in PHS. This suggested that the PHS could be retained on the surface where it is applied for comparatively longer period. With kind participation from three of our previous volunteers, we verified the above possibility through a similar thumb printing experiment as described earlier. We took the thumb impressions of these volunteers before sanitization and at different time intervals (0, 30 and 60 min) after sanitization with either standard hand sanitizer (+ ve control) or PHS (Fig. 2b). We observed that thumb prints taken immediately after sanitization did not show any microbial load for both standard and PHS (Fig. 2b panels (ii, vi, x)). However, colonies started getting visible in the prints taken after 30 min of sanitization. Visible comparison showed that these colonies were more numerous in the prints from thumbs sanitized with standard sanitizer (Fig. 2b panels (iii, vii, xi)). The microbial load was within check even after 60 min of sanitization with PHS. But, thumb prints taken after 60 min of sanitization with standard sanitizer, showed quite an increase in microbial load (Fig. 2b panels (iv, viii, xii)). Taken together, these observations proved the prolonged retention of PHS on the surface it is applied and its consequent prolonged anti-microbial activity.

### PHS has higher anti-microbial potency than standard hand sanitizer

**Fig. 3:**
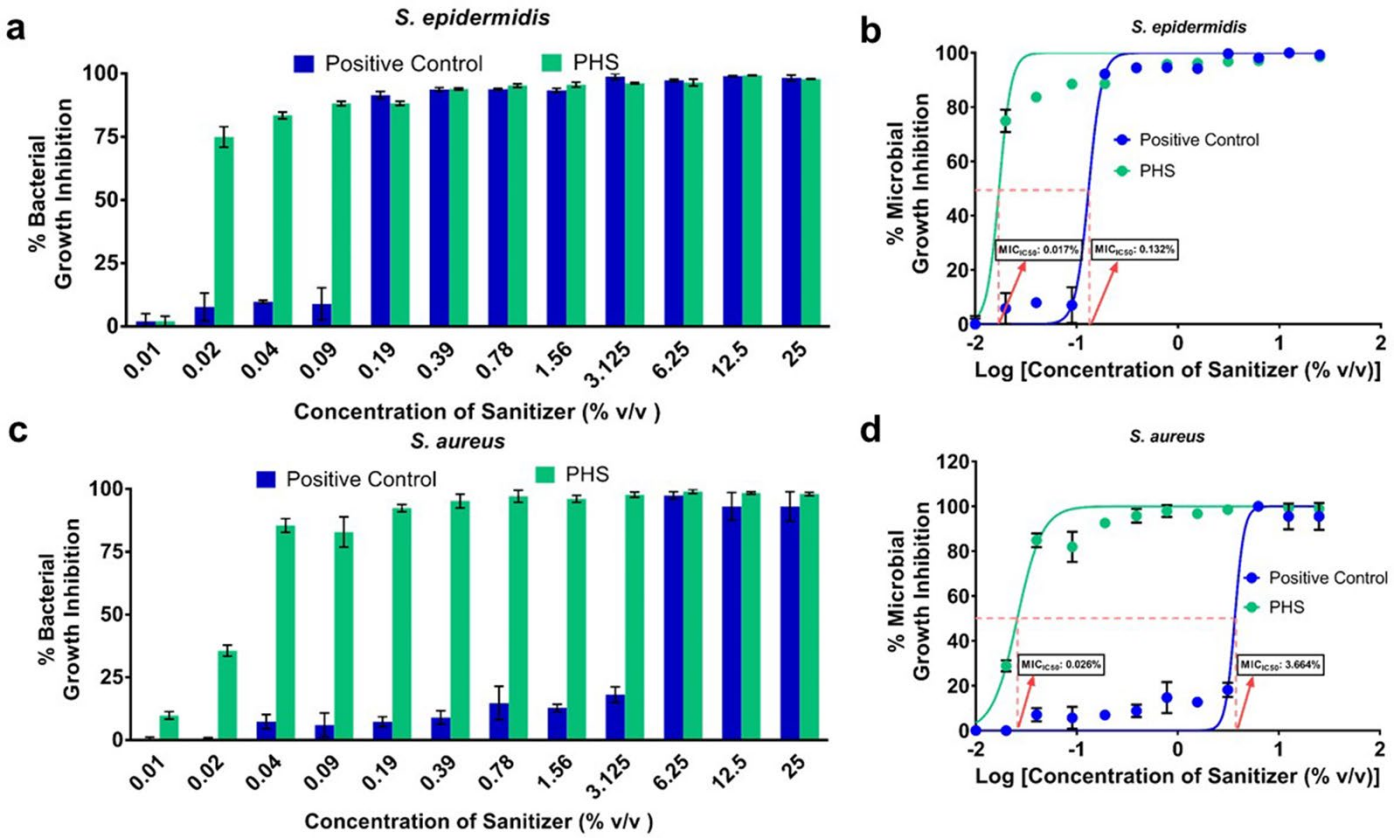
Enhanced anti-bacterial potency of Patanjali Hand Sanitizer (PHS). **a**, **c** Grouped column graphs representing comparisons between % bacterial growth inhibition exhibited by the standard hand sanitizer (used as positive control) and PHS against *S. epidermidis* (**a**) and *S. aureus* (**c**). **b**, **d** Anti-bacterial potencies of the standard hand sanitizer (positive control) and PHS against *S. epidermidis* (**b**) and *S. aureus* (**d**) are represented as dose response curves along with the calculated MIC_50_ values (concentrations at which either of the hand sanitizers is capable of 50% bacterial growth inhibition)

To assess the functional efficacy of PHS, we determined the inhibitory concentrations of PHS for *S. epidermidis* and *S. aureus* through micro-broth dilution method at which it showed 50% inhibition of the bacterial growth and referred to these values as MIC_50_. At 0.02% v/v dilution, PHS inhibited 74.90 ± 2.33% growth in *S. epidermidis*, while at same concentration the standard hand sanitizer exhibited 7.76 ± 3.16% growth inhibition (Fig. 3a). MIC_50_ of PHS against *S. epidermidis* was 0.017% v/v while that of the standard sanitizer was 0.132% v/v (Fig. 3b). Hill slope of 7.196 for PHS versus 6.716 for standard sanitizer clearly shows the remarkably improved anti-bacterial potency of the former. Likewise, at 0.04% v/v concentration, PHS inhibited 85.53 ± 1.58% while the standard hand sanitizer inhibited 7.41 ± 1.65% of *S. aureus* growth (Fig. 3c). Calculated MIC_50_ values for PHS and the standard sanitizer for *S. aureus* are 0.026% and 3.664% v/v, respectively (Fig. 3d). As with *S. epidermidis*, the hill slopes of 3.523 and 9.425 respectively for PHS and standard in case of *S. aureus* corroborate with the observation for *S. epidermidis* that anti-bacterial potentials of PHS are better than the standard hand sanitizer used for the study.

### PHS is equally safe as the commercially available hand sanitizer

**Fig. 4:**
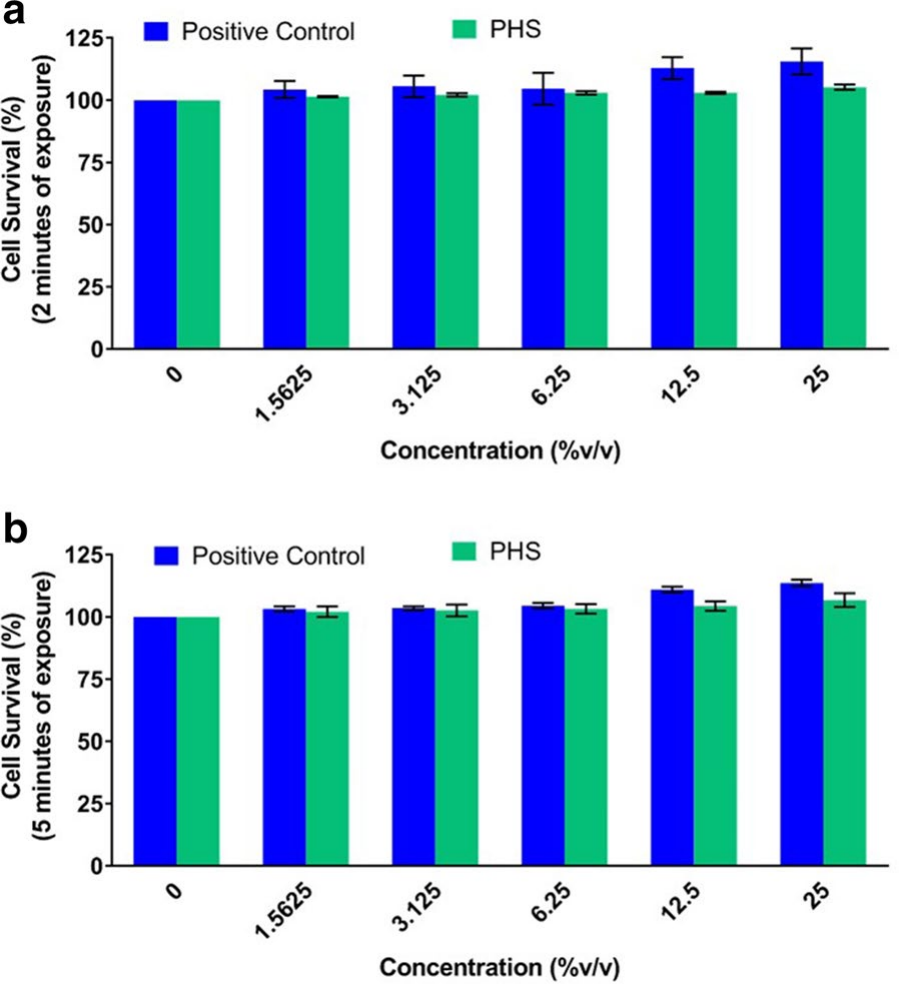
PHS is safe to be used as a hand sanitizer. **a**, **b** Grouped column graphs showing comparative cytosafeties of treating human squamous epithelial cells with standard hand sanitizer (positive control) and PHS for 2 (**a**) and 5 (**b**) min

With given COVID-19 pandemic, application of hand sanitizer has almost become a ritual. Endorsed by various standard operating protocols issued by health ministries all over the world, extensive hand sanitization has become an integral part of life. This means our hands getting exposed to sanitizers more than ever and it is not restricted to clinical or laboratory set-ups only. This increases the likelihood of our skin cells experiencing prolonged exposure to chemicals foreign to them. Therefore, it is pertinent to ensure the cytosafety of the hand sanitizer we are using, which we ensured through standard alamer blue cell viability assay using human squamous epithelial skin cell line (A-431). We incubated the cells in different dilutions of standard sanitizer and PHS for either 2 min or 5 min before replacing the sanitizer exposure with regular growth medium for 24 h, when the cell viability was assessed. As expected, we did not observe any cytotoxic effect from standard sanitizer, nor did we find PHS to be toxic (Fig. 4a, b). So, PHS was found to be equally safe as the standard hand sanitizer for skin the cells.

### PHS imparts bactericidal effect through disruption of bacterial cell membrane

**Fig. 5:**
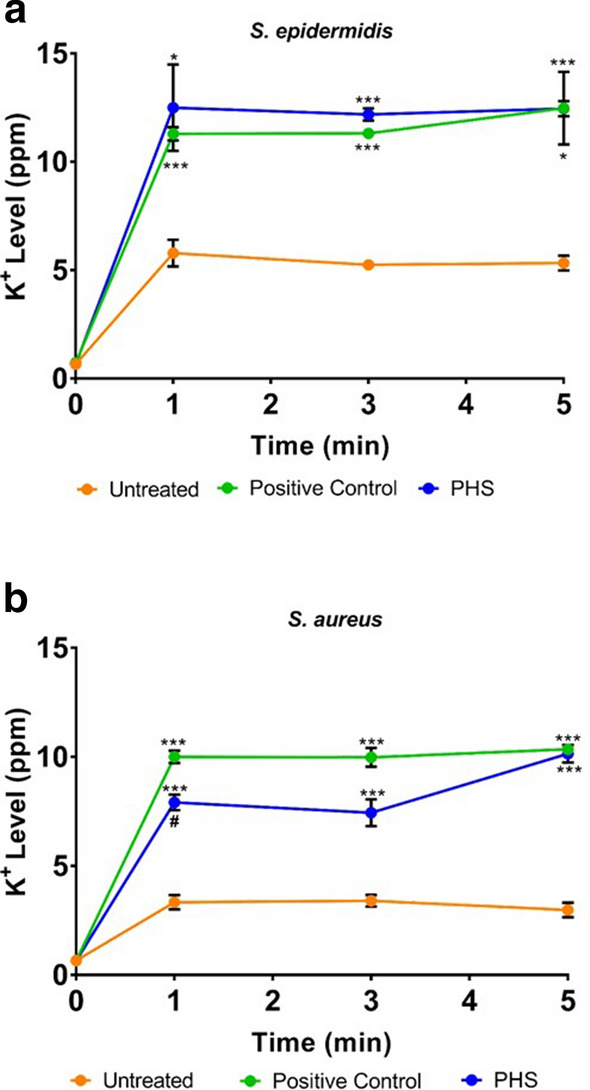
Mode of anti-bacterial activity of PHS. **a**, **b** Bacterial membrane disruption by PHS is evaluated through potassium (K^+^) ion efflux in *S. epidermidis* (**a**) and *S. aureus* (**b**) measured as parts per million (ppm) of K^+^ ions in the medium and represented comparatively over a period of 5 min post-treatment with standard hand sanitizer (positive control) and PHS

The standard being an alcohol based sanitizer, killed bacteria through disrupting the bacterial cell membrane as evident from potassium (K^+^) ion leakage from both *S. epidermidis* and *S. aureus* cells (Fig. 5a, b). When we estimated the K^+^ ion leakage for PHS treated *S. epidermidis* and *S. aureus* cells, observations were comparable to those of standard sanitizer, thus, suggesting that the bactericidal effect of PHS is due to its ability to disrupt bacterial cell membranes.

## Discussion

To meet the growing demand for hand sanitizers, PHS was formulated keeping the cost-effectiveness and safety as the two guiding factors. PHS is comparable to the standard hand sanitizer in all respects with some added advantages. PHS, being a gel based hand sanitizer, does not dry up the skin from frequent hand sanitization, a feature, definitely, with a big plus under current pandemic situation where, very frequent hand sanitization is almost becoming a rule. The *Aloe vera* gel present in PHS makes it soothing for the skin and helps in moisturing the hands, and kind of doubles up as a hand moisturizer. Besides, *A. vera* is known for its anti-bacterial, anti-fungal and anti-viral properties (Athiban et al. 2012; Rezazadeh et al. 2016).

COVID-19 pandemic has spirally increased the sales of hand sanitizers. The demand for the product has increased to such an extent that supermarkets and pharmaceutical stores have limited the number an individual can buy at a time. Currently, there is a huge gap in the demand–supply line of hand sanitizers in the world. Naturally, the prices of hand sanitizers have witnessed a surge across several international markets, due to its swelling demand. Several market players are actually deploying their manufacturing proficiency and amenities to address this shortfall; Dow Europe and Ineous, the leading polymer manufacturers, being one of them. In an attempt to cope with this scarcity, the Government of India has declared hand sanitizers, as one of the essential commodities, putting a cap on its price to discourage black marketing (https://www.globenewswire.com/news-release/2020/04/15/2016141/0/en/Hand-Sanitizer-Market-To-Reach-USD-7-32-Billion-By-2027-Reports-and-Data.html). Recently, to meet the surging demand for hand sanitizers, governments in many countries also adopted rationing of this product (https://www.beckershospitalreview.com/quality/how-much-hand-sanitizer-is-enough.html).

Depending on the size of the hands, 1.5 to 3 ml of sanitizer is required for effective sanitization of the hands. The recommended frequency of hand sanitization, as laid down by various standard operating protocols from different health organizations and ministries, is once per hour under the current pandemic condition (https://www.prudentialuniforms.com/blog/wait-often-sanitize-hands/). Thus, nearly, 20 to 50 ml of hand sanitizer is required per person per day. However, a sanitizer that is retained longer on the hands after application is likely to provide protection from germs longer, in turn, reducing the consumption. This will automatically reduce the demand and help in relaxing the current strain on supply. Moreover, the standard hand sanitizer used in this study (and other similar sanitizers) cost around 600 to 700 INR for 500 ml, while, PHS costs around 120 INR for a similar pack size. Thus, taking the functional efficacy and the price together into consideration, PHS is ~ 4.3 times more cost effective than the standard hand sanitizer. This market analysis recognizes the commercial potentials of PHS, with a clear humanitarian vision of helping mankind during this pandemic, amidst a diminishing global economy.

We have reported in this study a hand sanitizer with anti-microbial efficacy comparable to that of one of the topmost hand sanitizers currently used under clinical set-ups. This new hand sanitizer has prolonged retention on the surface where it is applied thus, having the potential to reduce the currently recommended frequency of hand sanitization. This, in turn, is likely to relax the gap in the demand–supply line of hand sanitizers. The functional efficacy, prolonged surface retentivity and market price of the reported hand sanitizer, taken together, appear to be a very cost-effective alternative to existing hand sanitizers.

## Data Availability

All data generated or analyzed during this study are included in this published article.

## Abbreviations

ATCC: American type culture collection
BAW: Bacterial load after wash
BBW: Bacterial load before wash
CDC: Center for disease control and prevention
CFU: Colony forming unit
COVID-19: Coronavirus disease of 2019
DMEM: Dulbecco's modified eagle medium
MHA: Muller Hilton agar
MIC: Minimum inhibitory concentration
PHS: Patanjali hand sanitizer
WHO: World Health Organization
